# Reported Diabetes Mellitus Prevalence Rates in the Colombia Healthcare System from 2009 to 2012: Analysis by Regions Using Data of the Official Information Sources

**DOI:** 10.1155/2015/946419

**Published:** 2015-10-01

**Authors:** Noël C. Barengo, Diana Carolina Tamayo

**Affiliations:** ^1^Observatorio de Diabetes de Colombia, Organización para la Excelencia de la Salud, Carrera 11A No. 94-76, Office 201, 110221 Bogotá, Colombia; ^2^HJELT Institute, University of Helsinki, Mannerheimintie 172, 000014 Helsinki, Finland

## Abstract

The objective of this study was to describe the reported diabetes mellitus (DM) prevalence rates of the 20–79-year-old population in Colombia from 2009 to 2012 reported by the healthcare system. Information on number of patients treated for DM was obtained by the Integral Information System of Social Protection (SISPRO), the registry of the Ministry of Health and Social Protection, and the High Cost Account (CAC), an organization to trace high expenditure diseases. From both sources age-standardized reported DM prevalence rates per 100.000 inhabitants from 2009 to 2012 were calculated. Whereas the reported DM prevalence rates of SISPRO revealed an increase from 964/100.000 inhabitants (2009) to 1398/100.000 inhabitants in 2012 (mean annual increase 141/100.000;* p* value: 0.001), the respective rates in the CAC register were 1082/100.000 (2009) and 1593/100.000 in 2012 (mean annual increase 165/100.000;* p* value: 0.026). The number of provinces reporting not less than 19% of the highest national reported DM prevalence rates (1593/100.000) increased from two in 2009 to ten in 2012. Apparently, the registries and the information retrieving system have been improved during 2009 and 2012, resulting in a greater capacity to identify and report DM cases by the healthcare system.

## 1. Introduction

Diabetes mellitus (DM) is one of the fastest growing public health problems imposing a high burden on DM patients and high financial burden on healthcare systems. Recent estimates of the International Diabetes Federation (IDF) predicted that the number of adults with diabetes is expected to rise worldwide from 366 million in 2011 to 552 million by 2030 [1]. The last Colombian National Health Survey in 2007 revealed a DM prevalence of 3.5% in the Colombian population 18–69 years of age according to participant self-report [2]. However, according to the IDF there were 2.1 million DM cases in Colombia corresponding to a DM prevalence of 7.05% in 2013 for the 20–79 year-old population [1].

It has to be kept in mind that there is a remarkable gap between people diagnosed with DM and those receiving treatment. It has been shown that only approximately 50% of the people diagnosed with DM receive medical care [3, 4]. Currently, no national register exists for DM patients in Colombia. However, for the last six years, two official registers have been developed in Colombia to track the number of DM—and other relevant diseases—cases that demand healthcare attention [5–7]. These registers use the available information provided by the healthcare providers and insurers. Nevertheless, no intent has yet been made to analyze the existing information in order to see whether there are changes in the access to DM treatment within the healthcare system during the past years. Furthermore, it is important for the primary healthcare system to monitor the number of DM patients receiving treatment on a regular basis to evaluate its capacity to detect new DM cases and to follow up whether it is capable to reduce the gap between diagnosed DM cases and those receiving medical care.

The aim of this study was to describe the reported DM prevalence rates of the 20–79-year-old population in Colombia during 2009 and 2012 reported by the official information sources of the healthcare system.

## 2. Material and Methods

Information on number of patients with DM as primary diagnosis in the population of 20–79 years of age was obtained by the Integral Information System of Social Protection (SISPRO) and the High Cost Account (CAC), an organization to trace high expenditure diseases. The SISPRO is a register developed by the Colombian Ministry of Health and Social Protection which offers consolidated data from the healthcare services demand in Colombia. All healthcare providers (hospitals and healthcare centers) are by law obliged to send information using the ICD-10 code of the primary diagnostics of all patients placed in the SISPRO register; thus, covers the entire Colombian population that demands services within Social Security Health System. The SISPRO register is fed by different information sources of healthcare service data after careful validation of each. Data submitted to the SISPRO register is subject to continuous quality control testing [5, 6]. The data received is cross-checked against other information sources (such as population census, national health surveys, or other administrative registers) before being integrated into the SISPRO register. If inconsistencies are detected, the data is sent back to the reporting institutions for revision and correction. In addition, according to the existing regulation several specific projects are carried out to improve primary data sources in order to guarantee a better quality of data received. The SISPRO registers the primary diagnostic code of the healthcare visit of both primary care and hospital care visits. Data on healthcare services provided to DM patients of the SISPRO for the years 2009–2012 were obtained in October 2013 using the ICD10 codes E100–E149, G590, G632, H280, H360, M142, N083, O240–O244, and O249. Many DM patients contact the primary healthcare system at very advanced stage of their disease presenting already complications of DM. These patients are usually registered with an ICD-10 code related to complications of DM as primary diagnostic code. Therefore, we have included these ICD-10 codes in our search to ensure that otherwise invisible cases of DM were included in the analysis. In addition, the ICD-10 differs between insulin-dependent DM (E10) and non-insulin-dependent DM (E11). However, since a large proportion of patients with type 2 diabetes are treated with insulin, they are coded as E10. In addition, the ICD-10 codes E12, E13, or E14 refer to unspecified DM. The consequence of this is that the ICD-10 codes do not allow a genuine and credible classification of clinical types of DM at least in Colombia.

The CAC is an agency established by the Colombian State in 2008 and managed by the healthcare insurance companies. The objective of the CAC is to monitor high cost illnesses within the Social Security Health System (SSHS) in order to develop mechanisms for financial management and to promote risk management in relation to high-cost illnesses [7, 8]. By law, the healthcare insurance companies must report all cases of DM, hypertension, and chronic kidney diseases that occur within their healthcare users. The CAC register covers 90% of the Colombian population. In this study, information of all identified cases of DM was received from CAC for the years 2009, 2010, 2011, and 2012 [9–12].

It is important to mention that both of the above mentioned registers only provide information about DM cases that have required healthcare attention. Thus, DM cases that have not been in contact with healthcare services do not enter the official registries. In addition, there is no data available in regard to the type of DM.

Information regarding population size and projections was retrieved from the National Administrative Department of Statistics (DANE). DANE is the organization that processes and shares the official Colombian population statistics [13]. DANE has also been considered as the official source of information about mortality and some health indicators in Colombian [14, 15].

### 2.1. Statistical Analysis

**Figure 1:**
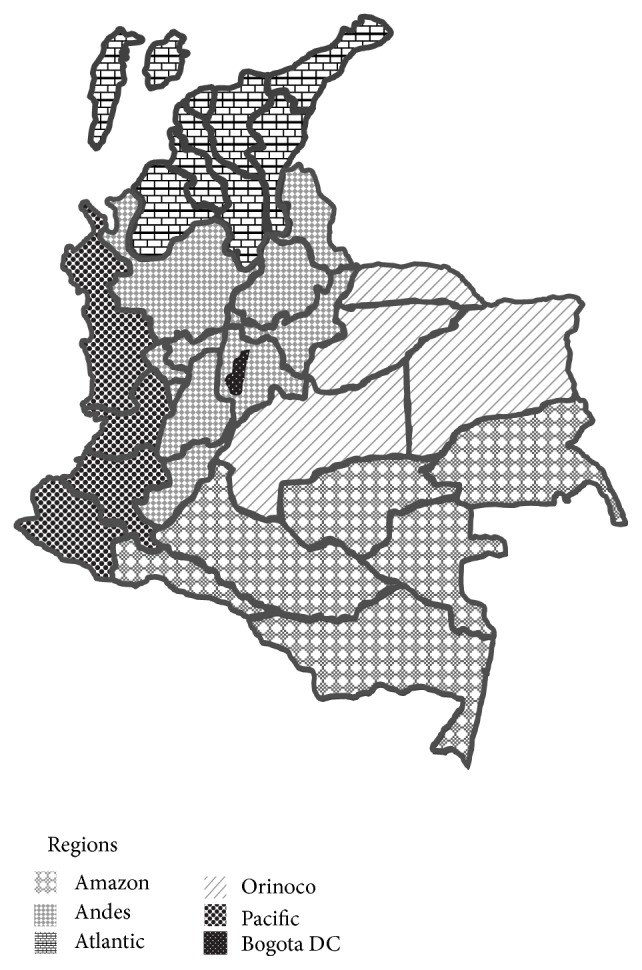
The six regions of Colombia and its provinces.

Age-standardized reported DM prevalence rates per 100.000 inhabitants for the population of 20–79 years of age from 2009 to 2012 were calculated separately for the SISPRO and the CAC registers as a quotient: The numerator was number of cases of DM in people 20–79 years of age in the SISPRO and, respectively, CAC register, whereas the number of inhabitants aged 20–79 years reported DANE population projections were used as the denominator. The EpiDat software version 4.0 was used to standardize the reported DM prevalence rates according to age using as reference population estimated for Colombia for 2010 according to United Nations population. The estimation of age-standardized reported DM prevalence rates for each of the six regions (Figure 1) corresponds to the average age-standardized rates calculated for the provinces in each region. The age-standardized reported DM prevalence rates are presented per 100.000 people for the age range 20–79 years.

The statistical analyses for time trends were performed with SPSS for Windows 14.0. They were tested first for linearity using Mantel-Haenszel test and for statistical significance the Chi-square test was used. The trends in prevalence were subsequently calculated using the linear regression model log⁡(PR) = *a* + *bt* + *e*, where PR refers to prevalence, *t* is time, *e* is the error term, and *a* and *b* are the regression coefficients estimated from the data. The change in prevalence per year at the time point *t* is a constant proportion, 100*b* percent of the prevalence at *t*. The estimated average yearly change in percent is exp⁡(*b*) − 1, which is approximately *b* for small changes. 100∗exp⁡(*b*) is presented in the tables. The level of statistical significance was set to 0.05.

**Figure 2:**
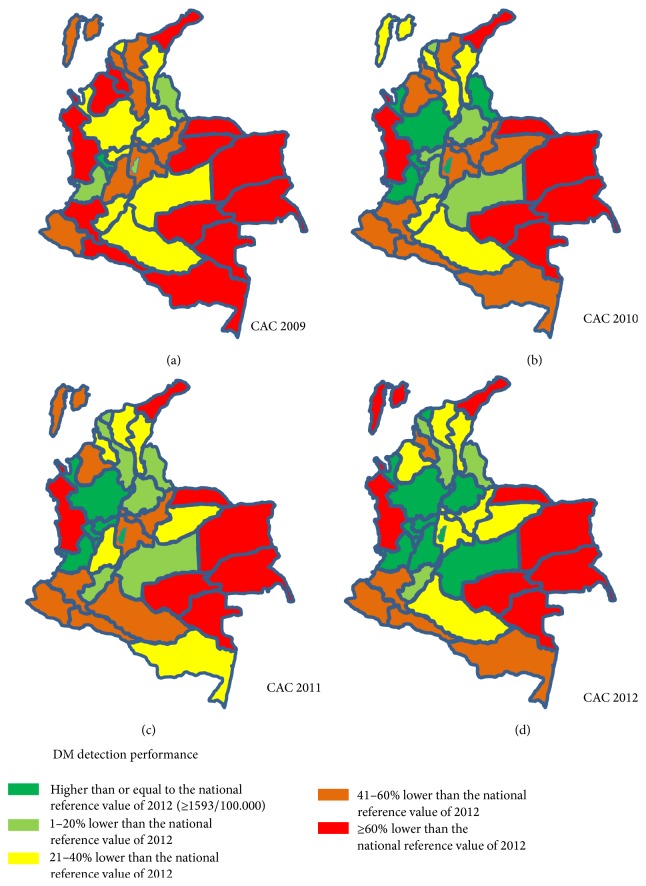
(a) Reported DM prevalence rates (CAC register) of the different provinces of Colombia in 2009 compared to the best national reported DM prevalence rate (1593/100.000). (b) Reported DM prevalence rates (CAC register) of the different provinces of Colombia in 2010 compared to the best national reported DM prevalence rate (1593/100.000). (c) Reported DM prevalence rates (CAC register) of the different provinces of Colombia in 2011 compared to the best national reported DM prevalence rate (1593/100.000). (d) Reported DM prevalence rates (CAC register) of the different provinces of Colombia in 2012 compared to the best national reported DM prevalence rate (1593/100.000).

Finally, for the maps presented in Figures 2(a), 2(b), 2(c), and 2(d), the annual reported DM prevalence rates of each province were categorized into five categories according to the performance in regard to the reported number of DM patients compared to the highest national reported DM prevalence rates (1593/100.000 in 2012) in the study period. The categories were as follows: (i) higher than or not more than 19% lower than the highest national value of 2012 (≥1592,6/100.000); (ii) 1–20% lower than the national value of 2012; (iii) 21–40% lower than the highest national value of 2012; (iv) 41–60% lower than the highest national value of 2012; and (v) ≥60% lower than the highest national value of 2012, respectively.

## 3. Results

The trends of reported DM prevalence rates in each region are presented in Table 1. Both registers showed a statistically significantly linear increase in reported DM prevalence rates per 100.000 people during 2009 and 2012. Whereas the reported DM prevalence rates of SISPRO revealed an increase from 964/100.000 inhabitants (2009) to 1398/100.000 inhabitants in 2012 (mean annual increase 141/100.000; *p* value: 0.001), the respective rates in the CAC register were 1082/100.000 (2009) and 1593/100.000 in 2012 (mean annual increase 165/100.000; *p* value: 0.026). The CAC register reported between 12% and 19% more DM cases/100.000 inhabitants compared to the rate provided by the SISPRO register. This difference did not statistically significantly change between 2009 and 2012.

Table 1 shows the age-adjusted reported DM prevalence rates per 100.000 inhabitants 20–79 years old in the different regions of Colombia according to the SISPRO and the CAC register during 2009 to 2012. The reported DM prevalence rates calculated from SISPRO reported a statistically significant increase in the Andes region (*p* value: 0.002), the region of Orinoco (*p* value: 0.039), and the Pacific region (*p* value: 0.020) during the time period, whereas the DM prevalence rates calculated from CAC register had a statistical significant linear increase in the age-adjusted reported DM prevalence rates in the regions Andes (*p* value: 0.022), Pacific region (*p* value: 0.004), and Bogota DC (*p* value: 0.035). Both registers identified more DM cases/100.000 inhabitants in 2012 compared to 2009. However, the annual increase in DM rate ranged from 26 to 153/100.000 people in the SISPRO register and from 25 to 203/100.000 inhabitants in the CAC register, respectively.

As during 2009 and 2012 the reported DM prevalence rates of CAC were regularly higher than the ones provided by SISPRO, the changes in the reported DM prevalence rates of the different provinces are only shown for the CAC register. Figures 2(a)–2(d) present the development of the DM cases reported in the CAC register in the different provinces of Colombia during 2009 and 2012 compared to the national reference value of 2012 (1593/100.000). Whereas 13 provinces reported DM prevalence rates 80% or lower than the national reference value in 2009, only eight provinces remained that far away from the national reference value in 2012. Mainly, the four provinces in east of the country, Choco at the Pacific coast, and La Guajira in the north-east upper corner of Colombia did not show any improvement in detecting DM cases. Whereas in 2009 the reported DM prevalence rates of 21 provinces were below 60% of the national reference, the respective number in 2012 was 13 provinces. The number of provinces reporting at least the national reported DM prevalence rates of 2012 increased from two in 2009 to ten in 2012.

## 4. Discussion

Our study showed that the registries and the information retrieving system of SISPRO and CAC have been improved during 2009 and 2012 resulting into a greater capacity to identify and report DM cases by the healthcare system. However, some regions and provinces showed a slower or no increase in the reported DM prevalence rates creating a future challenge for the official healthcare information system of Colombia.

There are several possible reasons for the findings of our study. First, the capacities of the registers to identify the DM cases may have improved due to implementation of different screening or early diagnosis activities within the healthcare providers to detect people with DM. For instance, many healthcare insurance companies have recently implemented different benchmark system to catch DM patients as early as possible to offer efficient treatment, whereas others may use diabetes risk factors scores combined with laboratory tests [16]. As there is a growing awareness of the burden of DM in Colombia, many DM screening programs will be developed in Colombia in the next years that may lead to a further increase in DM rates 13 detected by the healthcare system [17]. Generally, once individuals are diagnosed, approximately 50% are treated with medication, both in developed and developing countries [18].

A second reasonable interpretation of our results may be that the diabetes incidence has increased during the past four years in Colombia. Both obesity and physical inactivity are two of the most important risk factors for DM. Given the increased prevalence of obesity between 2005 and 2010 in the Colombian adult population combined with low levels of physical activity this may be a reasonable explanation [19, 20]. The National Survey of the Nutritional Situation (ENSIN) of 2010 revealed that 35% of women and 34% of men 18–64 years of age were overweight. The corresponding prevalence for obesity was 20% (women) and 11.5% (men). Furthermore, only every second 18–64-year-old adult reached the current physical activity recommendations of 30 minutes of daily moderate intensity physical activity [20, 21]. Thus, the unfavorable development of the major risk factors of DM may be reflected in the increase in the DM rates of people with DM in the healthcare system. However, we would like to point out that the data present here is not a study on the true prevalence of DM in Colombia but rather about the capacity to report DM cases by the healthcare system. According to available evidence, every second a diabetes case has not been diagnosed. The government has put an emphasis on improving these registries in the past years obliging the healthcare providers to improve disease reporting by a series of new legislation. Thus, we think that the increase in the reported DM prevalence rates can rather be explained by the improved capacity of the healthcare system to detect previously unknown DM cases than an increasing prevalence of DM.

It has to be pointed out that the observed increase in reported DM cases in the Colombian population may be related to a better and easier access or higher demand to healthcare for DM patients leading to higher DM rates recorded in the reporting system. Furthermore, we hypothesize that the difference observed between the regions in regard to reported DM prevalence rates may be due to the geographical distances from the more developed urban centers. Probably, the access to healthcare services is more difficult and the healthcare provider's information systems may have poorer development compared to the more developed regions of the country.

The IDF reported a DM prevalence of 7.05% in Colombia [1]. Thus, there is a remarkable gap between the IDF estimation and the DM prevalence found by the Colombian National Health Survey [2] in 2007 (3.59%). Besides, considering that there are about 800 000 known DM patients in Colombia, it is of great concern that only 50% of them get admitted in the healthcare system.

Naturally, our study has some limitations. The data base of SISPRO only registers the primary diagnostic code leading to an underestimation of DM cases as some patients may have DM as underlying condition to other diseases. In addition, our analysis is restricted to the DM cases officially reported and does not reflect the true prevalence of DM rather than the DM cases that have been in contact with the healthcare system. However, the objective of our study was not to estimate the true DM prevalence but to report the DM prevalence rates of the official information sources of the healthcare system. Finally, the DM type and the treatment profile that these registered patients are receiving are not described by these sources. In any case, these two registers are the only available sources of information in regard to the number of treated DM patients in Colombia as the country does not count with an official register for DM1 or DM2 at the present moment.

## 5. Conclusions

The official information sources to track DM patients have improved their ability to detect and record DM patients in Colombia. However, many regions are still lacking behind the national reference showing lower DM detection rates. The future challenge for the Colombian healthcare system is to identify DM patients as early as possible and provide equal access to treatment as early as possible to decrease the burden of DM.

## Figures and Tables

**Table 1:** Age-adjusted reported DM prevalence rates per 100 000 inhabitants in the different regions of Colombia according to the SISPRO register during 2009 to 2012.

Region	SISPRO				Annual increase (%)	*p* value	CAC				Annual increase (%)	*p* value
	2009	2010	2011	2012			2009	2010	2011	2012		
Amazon	428	507	504	517	*26 *	*0.170 *	516	629	587	612	25	0.362
Andes	991	1148	1299	1415	*142 *	*0.002 *	1159	1411	1500	1643	154	0.022
Atlantic	661	726	779	1038	*118 *	*0.075 *	733	949	1006	1026	94	0.101
Orinoco	552	651	761	773	*77 *	*0.039 *	509	755	854	898	127	0.060
Pacific	544	696	855	906	*125 *	*0.020 *	762	899	1049	1144	130	0.004
Bogota DC	1117	1502	1448	1645	*153 *	*0.116 *	1269	1634	1759	1905	203	0.035

^∗^ *p* value for time trend.
